# Initial Geometrical Templates with Parameter Sets for Active Contour on Skin Cancer Boundary Segmentation

**DOI:** 10.1155/2021/9528460

**Published:** 2021-08-03

**Authors:** Prachya Bumrungkun, Kosin Chamnongthai, Wisarn Patchoo

**Affiliations:** ^1^Department of Electronic and Telecommunication Engineering, Faculty of Engineering, King Mongkut's University of Technology Thonburi, 126 Pracha Uthit Road, Bangmod, Thung Khru, Bangkok 10140, Thailand; ^2^School of Engineering, Bangkok University, Bangkok 10110, Thailand

## Abstract

For active-contour-based surgery systems, the success of skin cancer boundary segmentation depends on the initialization point of the snake model, which is a task originally performed by skillful experts, and on the parameters set for the algorithms of active contour. This paper proposes initial geometrical templates and parameter sets for the active contour on skin cancer boundary segmentation. To establish initial geometrical templates and parameter sets for the active contour, first, template candidates, which are geometrically designed by users in advance, are simply calculated based on similarity with a skin cancer boundary, and the candidate with the least difference is selected as an initial template. Initially, all candidate templates are performed before the test with some selected skin cancer samples by randomly varying needed parameters to determine parameter sets for each template. The parameter set is therefore implicitly selected as the suitable set with the selected initial template. Experiments with 227 skin cancer samples were performed based on our proposed initial templates and parameter sets, and the results show 99.46% accuracy, 97.43% sensitivity, and 99.87% specificity approximately in which accuracy, sensitivity, and specificity were improved by 0.26%, 0.36%, and 0.26%, respectively, compared with the conventional method.

## 1. Introduction

According to a WHO's (World Health Organization) report in 2016 [1], cancer is the worst death cause for humans. Among cancer types including breast, cervical, and lung, skin cancer was ranked as number 19 of human death causes, and the number of patients dramatically increase by approximately 1.7 million in 2016 [1] due to stronger ultraviolet (UV) in recent years. Among several approaches to cure skin cancer including surgery, radiation, and photodynamic therapy, surgery comprising Mohs microsurgery, laser surgery, and electrodesiccation and curettage are currently widely accepted as effective methods with less pain. However, surgery basically depends on skillful medical doctors who are limited in number, and treatment is normally costly [2] such that the existence of an automatic skin cancer surgery system would be useful to assist medical doctors curing patients.

One of the important fundamental functions of the automatic skin cancer surgery system is skin cancer boundary segmentation [3]; so many researchers are trying to focus on the research problem of segmentation of the skin cancer boundary to ensure the success of automatic surgery. In fact, the skin cancer boundary complicatedly consists of many tiny curves and angles with low contrast in some parts. It truly becomes difficult to accurately segment the boundary for automatic skin cancer surgery. In case that the segmentation is not properly performed, the skin cancer is not completely removed due to reduced segmentation, and the cancer may subsequently spread throughout the entire body. Thus, some neighboring normal flesh is removed with the skin cancer as buffer because the medical doctor intends to ensure that all cancer is removed. Therefore, if automatic segmentation efficiently functions, it ensures that all of the skin cancer is removed, and the pain caused by removing some neighboring flesh needlessly is simultaneously eased.

Previous research works of segmentation, especially those related to automatic skin cancer surgery, could be divided into a couple approaches that are supervised and unsupervised. The first group of supervised-based approaches [4–7] analyzed images of skin cancer and utilized existing image processing and machine-learning tools to segment the boundary of skin cancers. Some of these studies [4] focused upon cancer detection and discuss the benefits and costs in terms of automatic cancer detection and assistance systems. However, to implement an automatic skin cancer surgery system, segmentation fundamentally becomes a crucial basic function and vitally requires high accuracy. Moreover, the aforementioned supervised methods are evaluated to yield good results in skin cancer segmentation and detection.

In another unsupervised approach, Castillejos et al. [8] proposed wavelet-transformed fuzzy algorithms for dermoscopic image segmentation. This method used feature extraction in wavelet transform space before proceeding to the segmentation process, and three-color channels (RGB space) in wavelet-transformed space gather the color channels via the nearest neighbor interpolation (NNI). This type of preprocess using existing mathematic tools and some machine learning algorithms that were discussed in the supervised approach is highly evaluated as good mathematic segmentation methods, but the bad cases with negative faults are critically outstanding [9, 10]. These models were acceptable in applications of skin cancer detection that decide the cancer boundaries. Although methods used for active contours simultaneously provide poorer results [9, 10], negative faults were found less often compared with the mentioned methods. This was the critical point for the automatic surgery system that medically requires segmenting skin cancer boundaries accurately since the bad segmentation cases were seriously regarded as negative results for the surgery. In this case, the skin cancer was not completely removed and ultimately not cured. In the subgroup of active contour usage, Munir et al. [11] recently proposed adaptive active contours based on the variable kernel with constant initialization. This mathematically incorporated a force term that pushed the contour towards the object boundary by using a regularization term that has taken into account the smoothness of the level set function and an edge term that helped to stop the contour at required boundaries. This system achieved high accuracy, but initialization is not completely automatic to date. On the other hand, Mogali et al. [12] proposed template-based active contours using a generalized active contour formalism for image segmentation based on shape templates, and the shape template is subjected to a restricted affine transformation (RAT), which allows for translation, rotation, and scaling. The segmentation functions excellently for any shapes. Kirimasthong et al. [13] proposed a method of automatic initialization of GVF-type snakes in ultrasound images of breast cancer. The method was proved to deal well with ultrasound images of breast cancer. Rodtook et al. [14] proposed an automatic initialization of active contours and a level set method in ultrasound images of breast abnormalities. The method successfully dealt with ultrasound images of malignant tumors, fibroadenomas, and cysts. Nevertheless, the complexity of automatic initialization remained as a problem in these mentioned methods for system implementation and to set parameters (weighting factors, iteration steps, etc.) for active contours depending on human skill [15] and it was not clearly reproducible.

The authors of this paper hence have focused on the research problems of automatic initialization and parameter setting for active contours. The initialization and parameter setting are considered in this paper to be improved in terms of human skill independence, simpleness, and reproducibility. Although deep learning tool as convolution neural network (CNN) is recently accepted as powerful for classification, it basically needs a huge number of samples for training, which may not be suitable for some medical problems. The authors first sought to perform experiments on skin cancer images using SVM and snake model using a semiautomated method [16]. It was confirmed to work well with some skin cancer samples but needed to be improved as a fully automatic method. The contribution of this paper hence is to create an algorithm to automatically initialize the active contour using a geometric template which is automatically selected from a group of geometric shape candidates based on some samples trained in advance. Since there exist many parameters in active contours which vitally influence convergence of the contours, parameters, which are matched with selected geometric template shapes, are automatically selected based on prior training of parameter sets. The active contours therefore can simply perform segmentation by the independence of any human skills.

This paper is constructed as follows: analysis of initialization and parameter setting for active contour-based segmentation is reported in Section 2. An overview of the imagined automatic surgery system and the proposed method are described in Section 3.  Section 4 demonstrates the experimental results using proposed templates and parameter sets. The discussion of the selected initial templates and parameter sets is explained in Section 5. Finally, the conclusion is presented in Section 6.

## 2. Analysis of Initialization and Parameter Setting for Active-Contour-Based Segmentation

**Figure 1:**
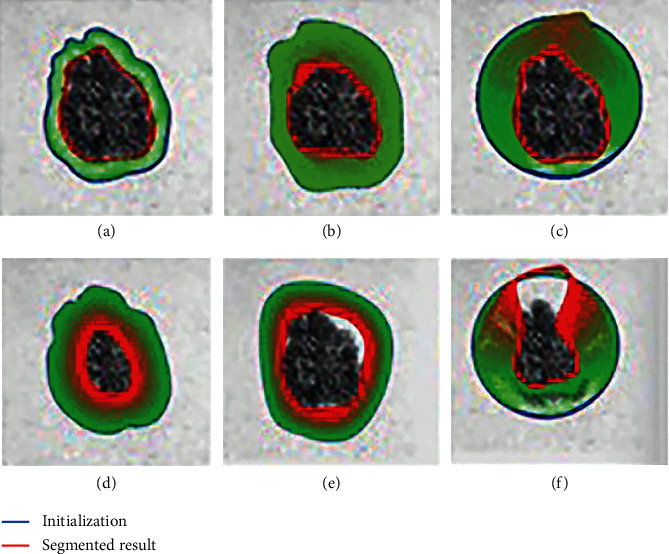
Comparison of different initialization and parameters. (a) Appropriate manual initialization with appropriate parameters. (b) Inappropriate manual initialization with appropriate parameters. (c) Geometrical initialization with appropriate parameters. (d) Appropriate manual initialization with inappropriate parameters. (e) Inappropriate manual initialization with inappropriate parameters. (f) Geometrical initialization with inappropriate parameters.

It is a fact that segmentation obtained by active contours is accepted as an excellent one, but it depends upon initialization and parameters based on human skill. If initialization is fit with the object shape, segmentation and active contours may be performed appropriately. As shown in Figure 1(a) where blue and red lines represent initialization manually performed by an expert and segmentation done by active contours, respectively, the segmentation result is observed to converge appropriately. In the opposite way, when the initialization is not fit with the object shape shown by a blue line, the red line representing segmentation is observed to converge inside the object, as shown in Figure 1(b). It is obvious that initialization is one of the crucial keys for active contours, and originally it depends on human skill. Although some researchers have achieved good results to create algorithms for automatic initialization using seed explosion [13, 14], complexity remains as important issue that will be discussed in this paper. The approach of conventional methods is basically based on random distribution which is regarded as a good way to cover any kinds of shapes. Observing the shape of appropriate initialization, it looks similar to the segmentation but closely bigger. It is also very difficult to create such kind of the shape which is similar to any shapes of the skin cancer, because it is initially unknown. Our solution in this paper is to scope numbers of candidate shapes using similar geometric ones. As shown by an example of circle in Figure 1(c), although the number of convergence loops observed by thickness of the red lines is accumulated more than the case of good initialization, as shown in Figure 1(a), it finally can converge in the similar level with the initialization done by an expert.

On the other hand, it is also observed that parameters such as weight factors, iteration steps, alpha, beta, kappa, wline, wedge, and wterm influence the segmentation results. For instance, appropriate parameters are set on those cases, as shown in the first row of Figure 1, and all initializations converge well except for the bad initialization, as shown in Figure 1(b). If inappropriate parameters are applied to those cases, segmentation results represented by red lines reveal incorrect convergence with many loops. Obviously, this means parameters are another key for active contours.

**Figure 2:**
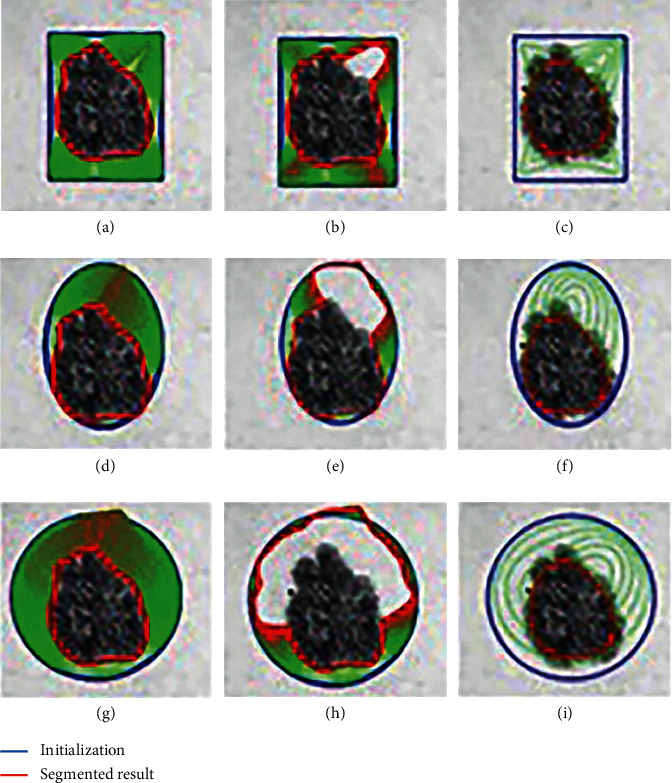
Comparison of snake initialized by geometric shapes with different parameters. (a), (d), and (g) Appropriate geometrical initialization with appropriate parameters. (b), (e), and (h) Appropriate geometrical initialization with too little convergence. (c), (f), and (i) Appropriate initialization with too much convergence.

As discussed earlier in Figure 1, initialization whose shape is similar to the skin cancer is the most preference for active contour segmentation, and an appropriate geometric shape is regarded as another choice used as a basic concept in this paper. Moreover, parameter setting is another crucial factor that controls active contour to converge appropriately. As shown in Figure 2, a skin cancer boundary is applied by different geometric templates which are rectangle, ellipse, and circle, as shown in row directions, respectively, and three parameter sets, which are assumed to be suitable for initialization, too less convergence, and too much convergence, are used in the column directions, respectively. These figures show the appropriate geometric shape with appropriate parameter set is preference, as shown in Figure 2(a), while others are unworkable based on inappropriate conditions of either initiation or parameter set, as shown in Figures 2(b)–2(i). This means both appropriate initiation and parameter set are really required in the implementation of fully automatic active contours, and the authors of this paper would find an algorithm to determine a geometric shape as initiation with a set of parameters which initially was fixed with the selected geometric shape.

Based on [17], the active contour or snake is defined as a deformable curve (**X**(**s**)=[**x**(**s**), **y**(**s**)]), where *s* ∈ [0,1]), and it is adjusted to minimize the energy (*E*) in the following equation:

(1) $$\begin{array}{c} E=\int_{0}^{1}\left(\alpha {\left|X^{\prime }\left(s\right)\right|}^{2}+\beta {\left|X^{\prime \prime }\left(s\right)\right|}^{2}+E_{\text{ext}}\left(X\left(s\right)\right)\right)\text{d}s, \end{array}$$

where *α* and *β* are weighting parameters corresponding to elasticity and stiffness of the snake, respectively, and they are assumed to be uniform for all. **X**′(*s*) and **X**″(*s*) are 1^st^ and 2^nd^ order derivative of **X**(*s*) with respect to *s*.

In operation, the snake curve represented by the first and second terms in (1), which exactly implies snake initialization, may be adjusted through the image spatial domain to reach the external energy *E*_ext_ representing image features. At that time, the total energy (*E*) should reach minima or even none, and the image boundary is regarded to be obtained. Since, normally, a shape of a skin cancer boundary is arbitrary, the snake operation sometimes cannot be performed accurately to segment the boundary due to some local minimum even logically reaching no total energy.

As the research theme of this paper mentioned earlier, the authors of this paper try to find a simple way to automatically initialize for practical snake operation for practical applications. Mathematically, geometric shapes can be approximated as candidates of the initialization, and the criteria of geometric shape selection should be an issue to discuss.

**Figure 3:**
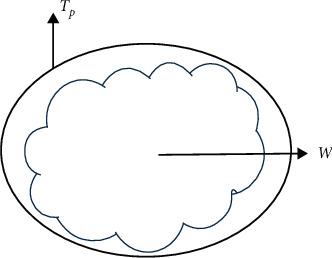
A geometric shape as initialization.

If some geometrical shapes (*T*_*i*_) are conceptually approximated in terms of template as initialization, a geometric shape (*T*_*P*_), which is selected based on the most similarity to the skin cancer image (*W*) from a group of candidate geometric shapes including circle, ellipse, triangle, rectangle, pentagon, hexagon, possibly differed the least compared with the skin cancer image, as shown in Figure 3. The geometrical template can be selected as follows:

(2) $$\begin{array}{c} T_{P}=\underset{i=1,2,\ldots ,n}{\min}T_{i}-W, \end{array}$$

where *n* is the number of template candidates.

The geometrical template candidates (*T*_*i*_), in which their scale is determined based on the maximum skeleton (*l*_max_) of the rough skin cancer boundary and their posture is varied by all angles (*θ*_*j*_) starting from an initial angle, can be expressed as follows:

(3) $$\begin{array}{c} T_{i}=T_{i}{\left\{l_{\max},\theta_{j}\right\}}_{j+t}^{2\pi +t}, \end{array}$$

where *t* and *j* are initial angle and angles around a point (*j*=0,1,2,…, 2*π*), respectively.

Therefore, the least difference between geometric initialization and skin cancer image is concluded as the condition for initialization determination in this paper.

## 3. Proposed Initialization and Parameter Setting Method for Active Contours

**Figure 4:**
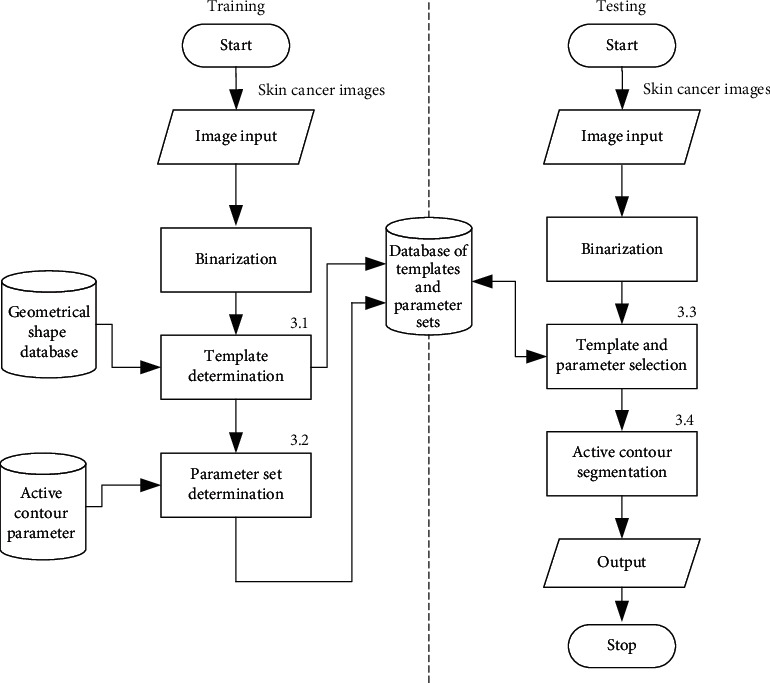
Flowchart of the proposed method. 3.1–3.4 represent subchapters explaining details of the processes.

In the implementation of the proposed method, some samples of skin cancer images need to be selected and trained to obtain candidate templates and their suitable parameters in the training state. As shown in the left column in the flowchart of Figure 4, the training state starts from inputting some known skin cancer images, performs fitting and voting for selection of candidate templates from geometrical shapes, and experiments those selected candidate templates with active-contour parameters for selecting parameter set for each candidate template. Those mentioned candidate templates with their suitable active-contour parameter sets would be stored in a database for usage in the testing state. The template and parameter determination processes will be explained in Sections 3.1 and 3.2, respectively.

In the testing state, suppose an unknown skin cancer image is inputted for segmentation, a suitable template with its parameter set will then be selected by searching in the database of templates and parameter sets trained with geometrical shapes and active-contour parameters in advance, and an active contour will finally segment the skin cancer based on the selected geometrical shape template with parameter set, as shown in the right column of the flowchart in Figure 4. The processes of template determination and active contour segmentation are mentioned in Sections 3.1, and 3.3, respectively.

### 3.1. Geometrical Template Determination

**Figure 5:**
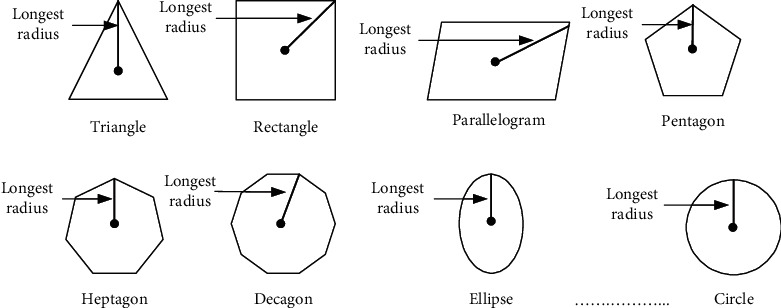
Geometrical shapes as template candidate.

**Figure 6:**
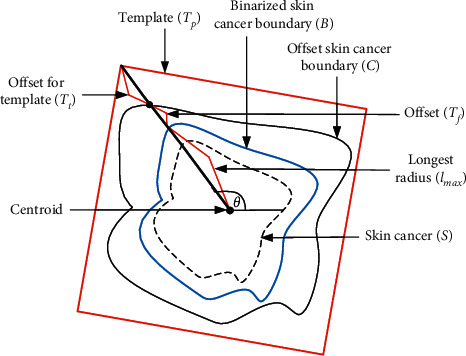
Mapping of geometrical shapes on a skin cancer boundary.

Normally, the best initialization for active contour is considered to fix the shape closest to the workpiece. Since the workpiece boundary is originally unknown, a way to determine initialization automatically is to estimate a rough boundary of the workpiece and utilize a geometrical shape as a template, which is closest to the rough boundary, as initialization. In the estimation of the rough boundary of the workpiece, which is assumed to be a skin cancer boundary, some preprocessing such as binarization process can be used to simply extract a border of rough skin cancer boundary which is the foreground of the image, and then offset should be added surrounding the extracted skin cancer boundary border to ensure the whole skin cancer boundary is picked up. In the utilization of geometrical shapes as candidate template for automatic initialization determination, all possible geometrical shapes, as shown in Figure 5, should be applied as a candidate to the rough skin cancer boundary with added offset, and all candidate geometrical shapes regarded as a template should be adjusted in scaling and rotation views to fit into the boundary. Conceptually, the centroid of a candidate geometrical template is first mapped in the same coordinates with the centroid of the rough skin cancer boundary, and differences between those mapped shapes are obtained in all scales and rotation angles of the template. In detail, the template first fixed its scale to be slightly bigger than the longest skeleton of the rough skin cancer boundary in the same centroid position, and then differences between the template and rough skin cancer boundary are obtained by rotating the candidate template in all angles, as shown in Figure 6. The algorithm of the mentioned steps is illustrated in Algorithm 1.

On the other hand, unknown skin cancer images performed segmentation in the testing state, as shown in the right column in Figure 6. In the testing state, all template candidates stored in the database of geometrical templates and parameter sets would be applied in a rough skin cancer boundary obtained by the binarization process. Like the processes in the training state, the candidate template with the least difference compared with the rough skin cancer boundary would be determined as the initial geometrical template for active contour operation, and the template and parameter set which has been trained and matched with the determined geometrical template are used in active contour segmentation in the next process.

**Figure 7:**
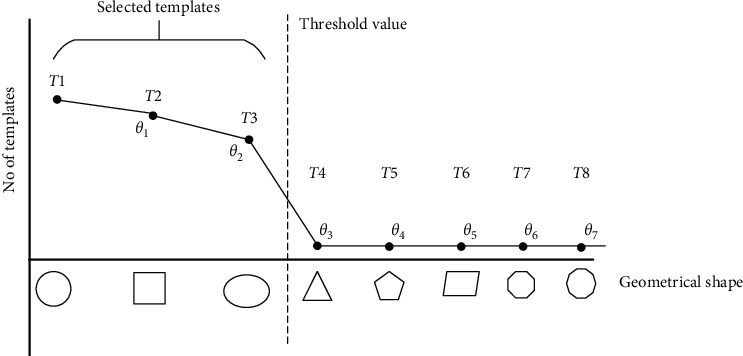
Determination of threshold value for screening geometrical shapes as template candidate.

In practice, it is almost impossible and redundant to find a suitable template from all existing geometrical shapes and find the difference between all possible templates with rough skin cancer boundary by continuously varying the templates in all angles. As shown in Figure 7, circle, rectangle, and ellipse should be selected as candidate templates, while other kinds of geometrical shapes, which are rarely used, should be excluded from the template candidate group. These rarely used geometrical shapes should be considered to delete in the training state. Therefore, a practical way to reduce redundancy of template determination from all possible geometrical shapes is recommended to limit the number of geometrical template candidates by high possibility based on some samples of skin cancer image as training state in advance. To determine high possibility geometrical shapes as templates stored in the geometrical shape database, as shown in the left column in Figure 4, some samples of skin cancer boundary image are manually selected for finding a threshold value. Consequently, those selected skin cancer samples are performed before testing based on the steps illustrated in Algorithm 1 for preprocessing, template selection, and template rotation starting from the 3^rd^, 9^th^, and 15^th^ lines, respectively. The number of determined geometrical templates would be simply counted, and a thresholding value [18] should be statistically determined for dividing between highly selected and less selected geometrical shapes. This means some geometrical templates, which are frequently determined as initialization template, would be screened as template candidates for storing in the geometrical shape database.

### 3.2. Determination of Parameter Set for Candidate Templates

It has been proved that parameters which are matched with initialization will help active contour to segment appropriately [19]. When geometrical shapes are selected as a template in the training state, parameters which are matched with those geometrical templates should be simultaneously performed before the test of active contour on some samples and determined in advance. These parameters which are the most matched in each geometrical template should be concluded as a parameter set of the geometrical template and would be utilized as initial parameters in the testing state.

In the training state, all parameters, which need to be set as initial ones, are listed up with varied ranges and steps, as shown by an example in Table 1. These ranges and steps of parameters would be used to vary in the pretest on some samples and concluded the best parameters by average as parameter set of the geometrical template. A computer program for finding parameter sets of all geometrical templates should be created following Algorithm 2. In Algorithm 2, a skin cancer image and geometrical templates are input in the 4^th^ and 8^th^ lines, respectively. Then, best parameter finding, parameter varying, and rotation are performed in 9^th^, 11^th^, and 13^th^, respectively.

In the training state, a geometrical template is performed mapping with some skin-cancer image samples while rotated in all 360 degrees; the best parameter sets for all samples are selected based on the least differences with samples, and the average of those best parameters are determined as parameter set of the template as shown in Algorithm 2. The parameter sets of all templates are stored in the database as fixed-parameter sets of templates and ready to be retrieved and used for the operation of active contour in the testing state.

### 3.3. Template Selection and How to Apply a Template to a Skin Cancer Image

In the testing state, when a skin cancer image is input, a geometrical template and parameter set will be selected, as shown on the right side of Figure 4. Like the training state, binarization is first performed to obtain a rough contour of the skin cancer image; then, an offset (*T*_*f*_) is added to the rough contour of skin cancer for ensuring to cover the whole skin cancer, and centroid and the longest radius (*l*_max_) of the workpiece are determined. Consequentially, mapping between the skin cancer image and candidate templates retrieved from the database is performed based on centroid and the longest radius and offset for template (*T*_*t*_) is added to ensure the template will cover the workpiece. Differences between templates and workpiece are obtained while the candidate templates are rotated around the centroid in all angles, and the best candidate template and the best rotation angle with the smallest difference between the candidate template and offset skin cancer boundary (*C*) are selected as initialization and will be applied for active contour. Simultaneously, a parameter set, which was fixed in the training state and stored in the database, would be retrieved determined by template. The algorithm is illustrated in Algorithm 3. In Algorithm 3, the rough contour of a skin cancer image is obtained in the 4^th^ line, and template mapping and selection are performed from the 7^th^ line.

### 3.4. Operation of Active Contour

Currently, there exist many kinds of active contours such as normal gradient vector flow [20], convolution vector flow [21], dynamic directional gradient vector flow [22], adaptive diffusion flow [23], and gradient vector flow (GVF) [24]. Users should consider selecting one of those active contours which is the most suitable for users' problems. The GVF has been selected in implementation and evaluation in this paper because it was designed and developed for the segmentation of complicate shapes and specially assumed to benefit varieties of medical applications.

It is well known that a couple of important keys for applying an active contour for image segmentation are initialization and parameters. In general, these are manually determined by experts based on the trial-and-error concept. The experts, who are going to tune up parameters to find the ones that are best matched with the images for segmentation, should well understand parameter characteristics for tuning suitably. These determined appropriate initialization and parameter set may contribute to energy minimization of active contour, especially for a local minimum.

This paper proposes initial geometrical templates for the automatic initialization of active contour. The geometrical template is first selected as the best matched with the skin cancer boundary image from a group of geometrical template candidates, and the selected template then performed mapping with the skin cancer boundary image in terms of scale and rotation. In addition, the parameter set, which was initially trained for each geometrical template, will be set with the initialization for active contour operation. To start running the operation of active contour, the mentioned geometrical template and parameter set are set in the program of active contour, as shown by an example in Table 1. When the active contour is executed, initialization will converge to the skin cancer image based on the energy minimization condition.

## 4. Experimental Results

This paper concentrates on the research problem of initial templates and parameter sets for initializing the active contour for skin cancer boundary segmentation and proposes a method for initial template and parameters setup. Since most of the medical image samples are sensitive and confidential, it is normally difficult to find samples for experiments and evaluation. To verify the effectiveness and evaluate the performance of the proposed method, the GVF snake algorithm was selected as active contour, and 227 images of skin cancer, as shown in the 2^nd^ column of Table 2, are used in experiments performed by the experiment set up, as shown in Table 3. Initial template candidates including circle, ellipse, and rectangle, which were automatically selected by our proposed method in terms of similarity with the skin cancer images, were used as candidate templates of initialization, and results of template selection and mapping are shown in the 3^rd^ column of Table 2. Initially, parameters for those templates for the GVF snake algorithm have been trained with 48 skin cancer image samples, and parameters sets for selected geometrical shape templates were determined based on the proposed method and concluded in Table 4. Experimental results done based on our proposed method and experts are shown in the 4^th^ and 5^th^ columns of Table 2, respectively. Finally, errors which are differences between the results of our proposed method and experts are shown in the 6^th^ column. Evaluation of our proposed method using geometrical shapes with parameter sets for automatic initialization can be concluded as 99.46% accuracy approximately with 13.61 sec per skin cancer image as computational time, while conventional methods [13, 14] achieved 96.41% and 99.20%, respectively, as shown in Table 5. On the other hand, sensitivity and specificity of conventional methods [13, 14] can be calculated [18] and concluded as 85.13% and 97.07%, and 99.83% and 99.16%, respectively, while the proposed method shows 97.43% and 99.87%, respectively, as shown in Table 5. Sensitivity (SEN), specificity (SPC), accuracy (ACC), and the Jaccard Index (JAC) can be calculated as follows [25].

Sensitivity:

(4) $$\begin{array}{c} \text{SEN}=\frac{\text{TP}}{\text{TP}+\text{FN}}. \end{array}$$

Specificity:

(5) $$\begin{array}{c} \text{SPC}=\frac{\text{TN}}{\text{TN}+\text{FP}}. \end{array}$$

Accuracy:

(6) $$\begin{array}{c} \text{ACC}=\frac{\text{TP}+\text{TN}}{\text{TP}+\text{TN}+\text{FP}+\text{FN}}. \end{array}$$

Jaccard index:

(7) $$\begin{array}{c} \text{JAC}=\frac{\text{TP}}{\left(\text{TP}+\text{FP}+\text{FN}\right)}, \end{array}$$

where TP, TN, FP, and FN are true positives, true negatives, false positives, and false negatives, respectively.

## 5. Discussion

This paper proposes initial geometrical templates and parameter sets as automatic initialization for the active contour on skin cancer boundary segmentation. The initial templates, which are geometrical shapes, such as circle, rectangle, ellipse, pentagon, are initially trained with some skin cancer image samples for template candidate determination. The parameter sets are accordingly established by varying all parameters and selecting the best parameter groups for each template.

In testing, candidate geometric shapes are compared with the input skin cancer image. The initial template is then determined by the least difference with the candidate initial templates, and the parameter set is used to initialize and run the active contour for the skin cancer boundary segmentation. Since normally medical image samples are not open in public due to personal information, it is difficult to collect a lot of samples. Moreover, the samples for evaluation based on conventional methods are not open in public, it is impossible to directly compare performance on the same samples. Therefore, the authors of this paper attempted to collect skin cancer image samples in a number, in which evaluation results can be relied on, to compare with the conventional methods. Experiments were performed based on our proposed method, and the performance of the proposed method achieves as high as 99.46% accuracy approximately, which is similar and slightly better than the conventional method [14]. However, in the case of skin cancer surgery, segmentation would originally be performed based on expert skill with added offset, and specificity, which represents false positive, should be importantly concerned. Our proposed method improves 0.26% specificity compared with conventional methods so that it is regarded to be useful for skin cancer segmentation. On the other hand, although modern and robust deep learning tool as CNN is currently considered as an excellent and powerful tool for classification and should be effectively applied in the skin cancer segmentation problem, it has been reported that active contour can perform comparably with CNN with less computation time [26].

Furthermore, since our basic concept is to approximate a similar geometrical shape as the initial template applied in the initialization of active contour, our initialization is originally apart from the skin cancer boundary. Mathematically, any shaped template which is close to the skin cancer boundary is the most suitable, but the algorithm may be complicate and time-consuming. The idea of using geometrical shape as a template for initialization is considered as a solution for implementation. As seen in the experimental results in Table 2, there exist obvious errors in skin cancer images, T4, T13, T18, T19, T39, T51, T59, T67, T88, T168, T169, T179, T185, T186, and T197. If template candidates are increased, some errors are partially solved. In fact, if a template is appropriately selected during the initialization, the snake algorithm should perform well. Basically, the concept of template implicitly includes comparing some errors with an initialized shape performed by experts. These errors should be traded off with computational cost. To improve initialization for active contour, learning and observing initialization done by skillful experts is recommended as future work.

For skin cancer boundary segmentation, our proposed method achieves 99.46% accuracy which includes both concave and convex results. As observed, most of the results are convex. However, some are partially included in concave, and most of them are tiny. In medical practice, miss-segmentation in convex is better than concave because all skin cancer cells are removed. The concave segmented boundaries are recovered by offset as a final boundary.

## 6. Conclusion

Automatic skin cancer image segmentation using active contour, which originally depended on skillful initialization done by experts, required practical automatic initialization with appropriate parameter sets. This paper proposed a method of initial geometrical templates with parameter sets for active contour on skin cancer boundary segmentation. Some skin cancer images were initially trained to evaluate and select geometrical shapes as candidate templates by mapping the geometrical shapes with skin cancer image samples based on the centroid and the longest radius and finding shapes with the least differences as candidate templates. These candidate templates were then used to perform active contour by possible parameters and then find a parameter set which performed the best active contour segmentation as a parameter set for each candidate template. In testing, these candidate templates performed mapping with a skin cancer image and rotating around the centroid in the same manner with training, and a geometrical template with the least difference with the skin cancer image was determined as the initial template for active contour. The determined geometrical template with the trained parameter set then was initialized for the active contour segment. Finally, the effectiveness of the proposed method has been evaluated by experiments with 227 skin cancer images.

## Figures and Tables

**Algorithm 1:**
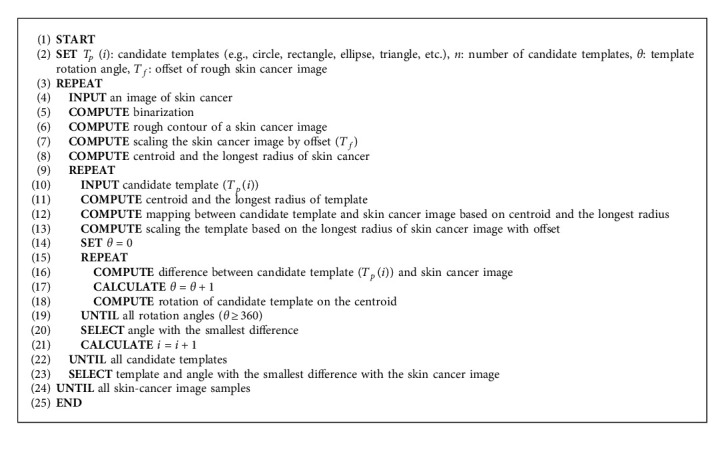
Template selection in training state.

**Algorithm 2:**
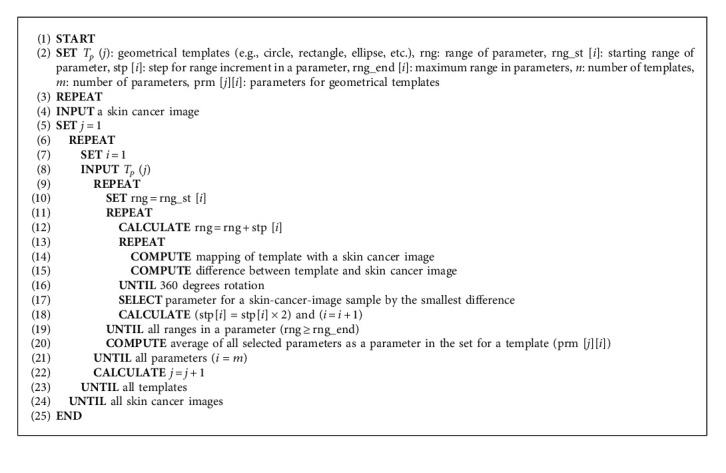
Parameter set determination for geometric templates.

**Algorithm 3:**
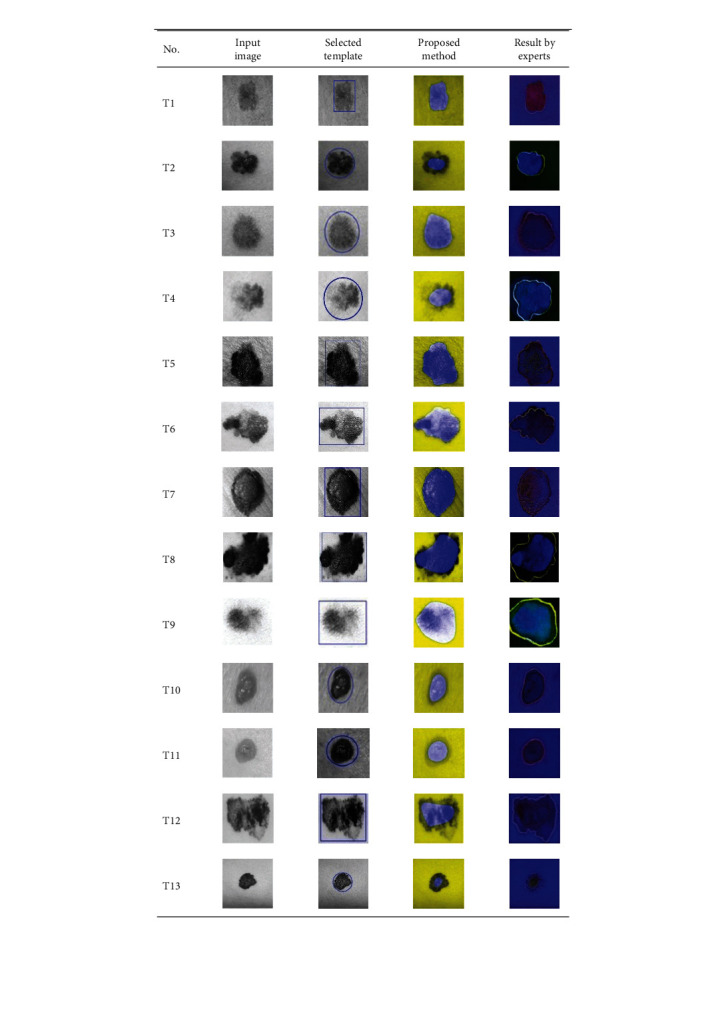
Template selection and applying the template to a skin cancer image.

**Table 1:** Parameters of active contour.

Parameter set	Range	Step
Interaction	100–400	100
nPoints	100–1000	100
Sigma1	1–10	1
Sigma2	1–20	5
Sigma3	0–1	1
Wline	0–0.01	0.01
Wedge	2–50	2
Wterm	0.01–100	10
Kappa	0–5	1
Alpha	0.02–7	0.2
Beta	0.01–2	0.01

**Table 2:** Experimental results of skin cancer segmentation.

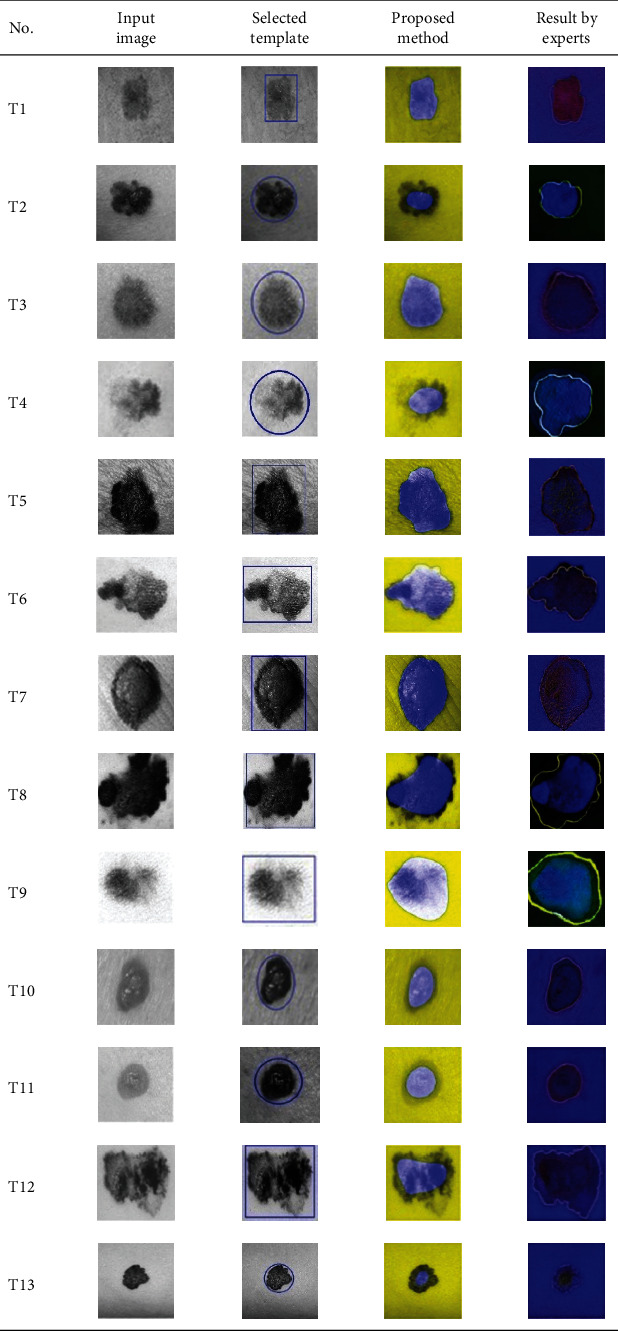
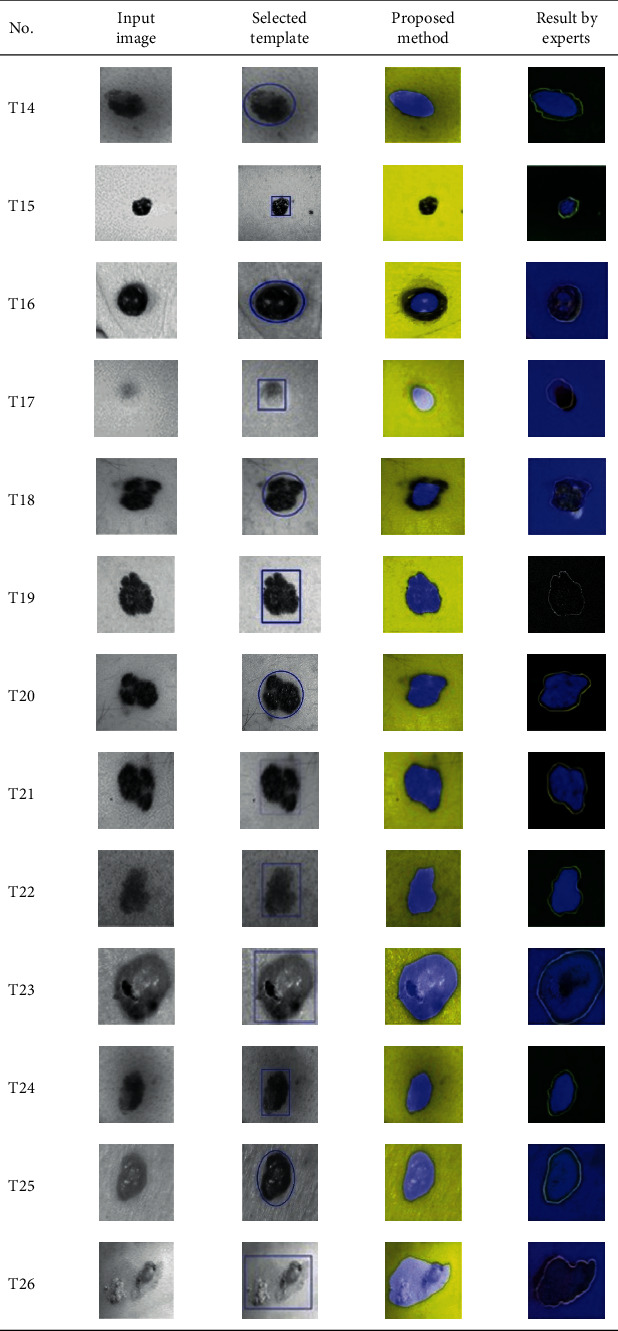
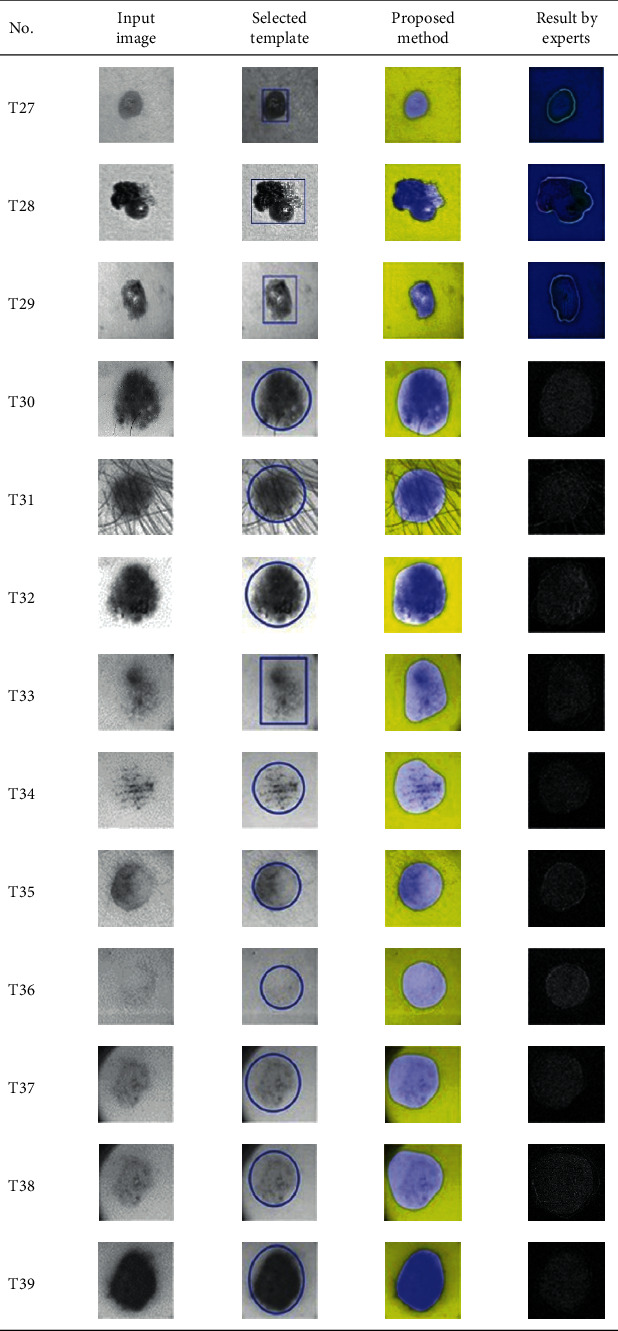
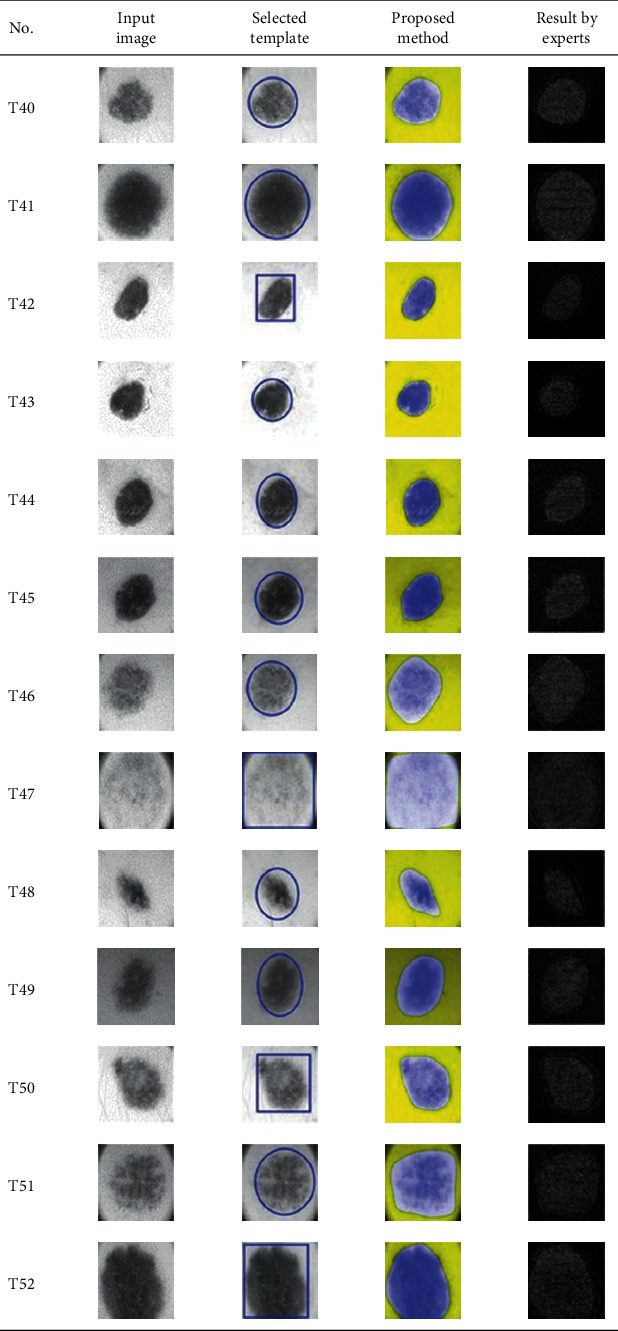
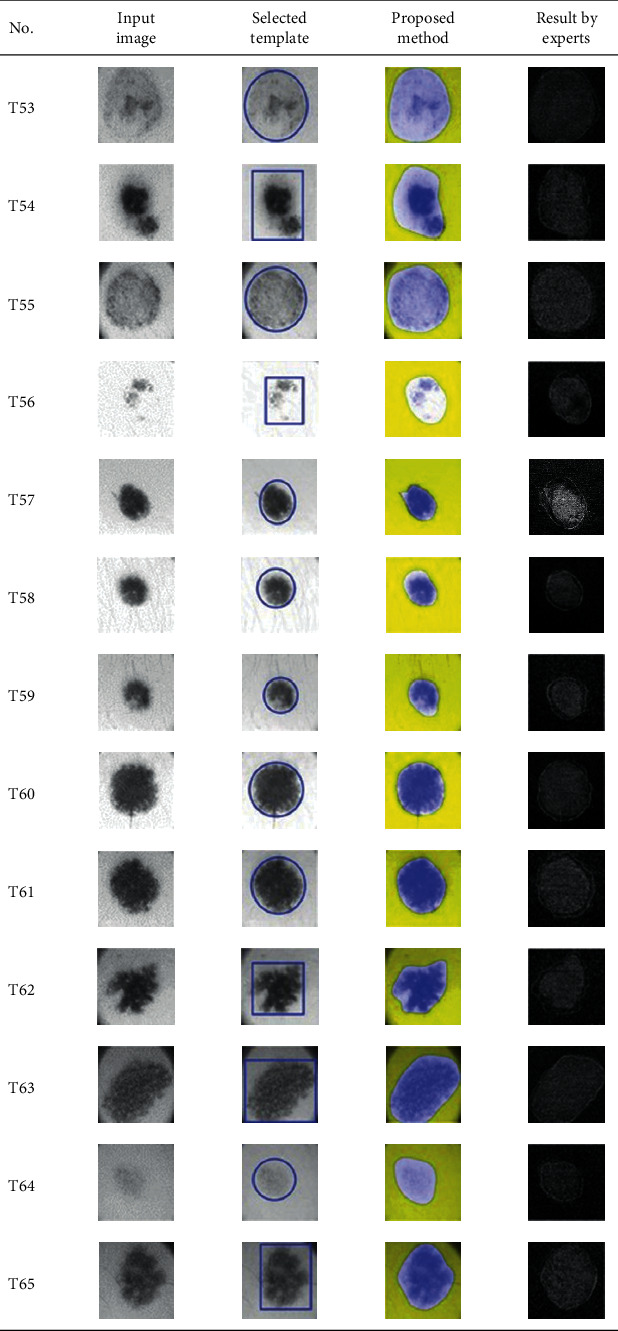
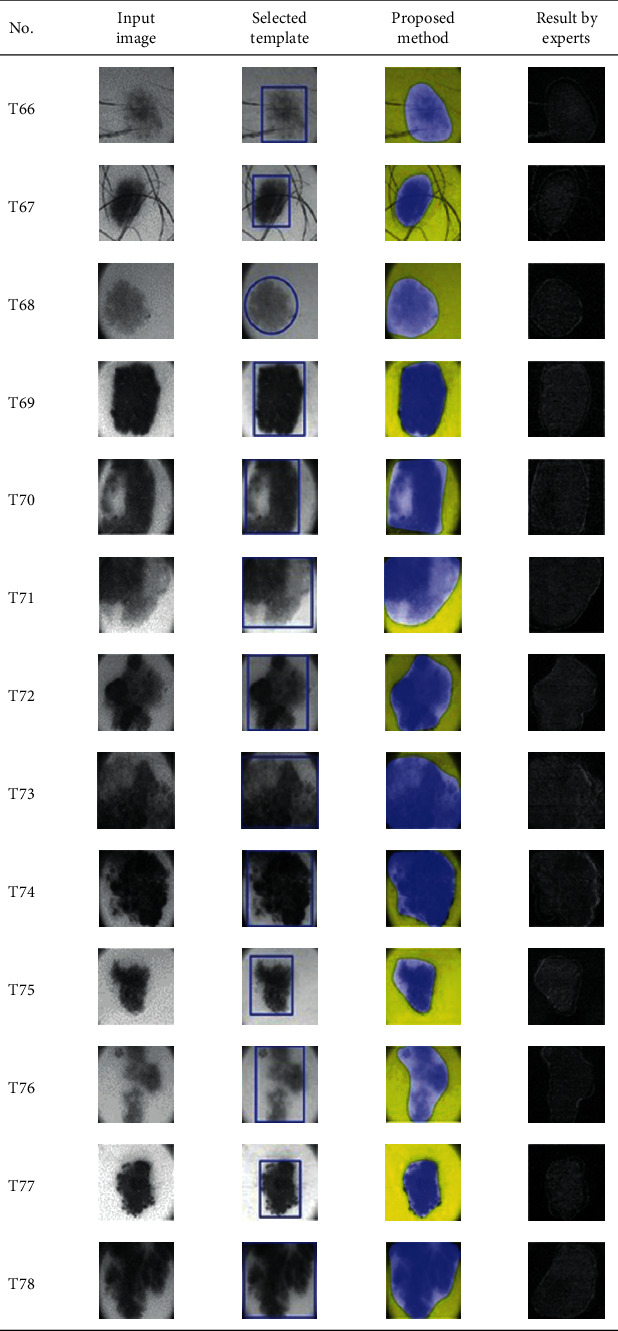
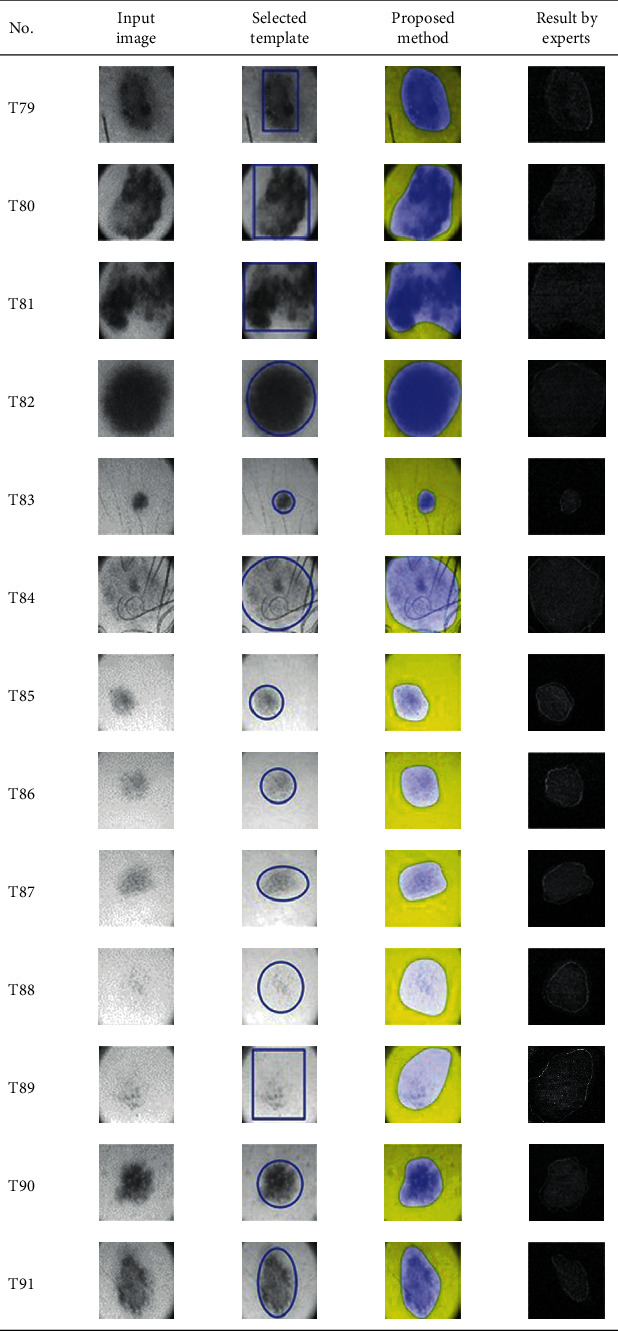
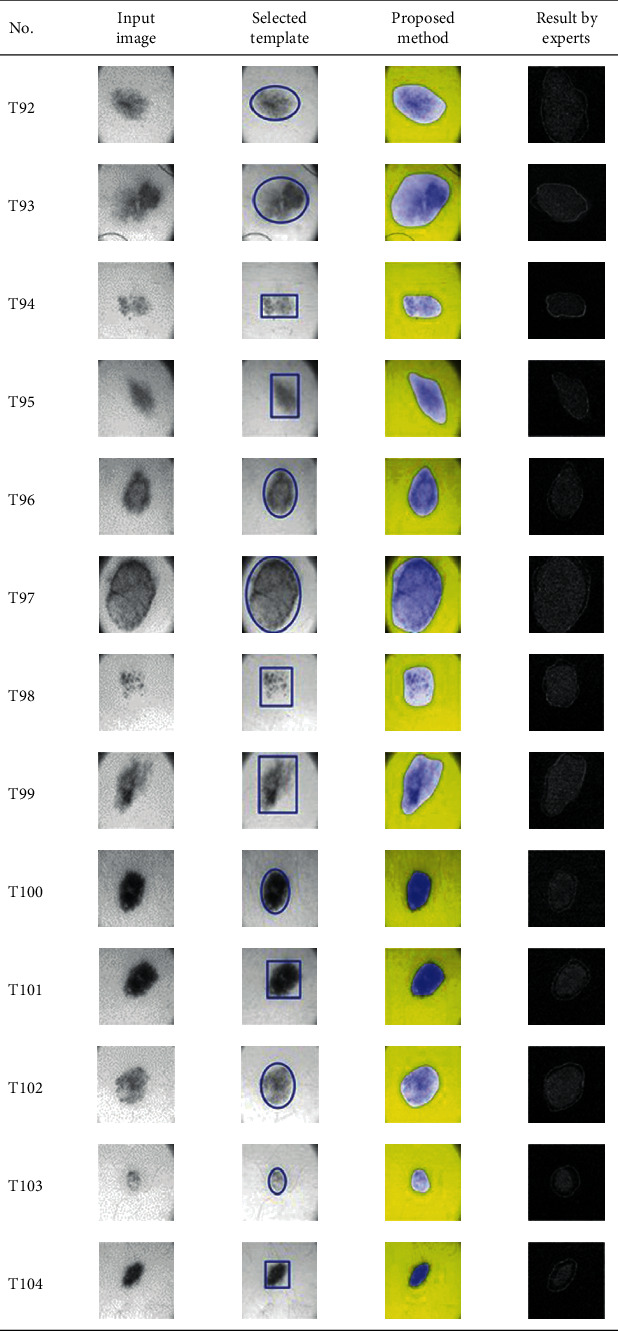
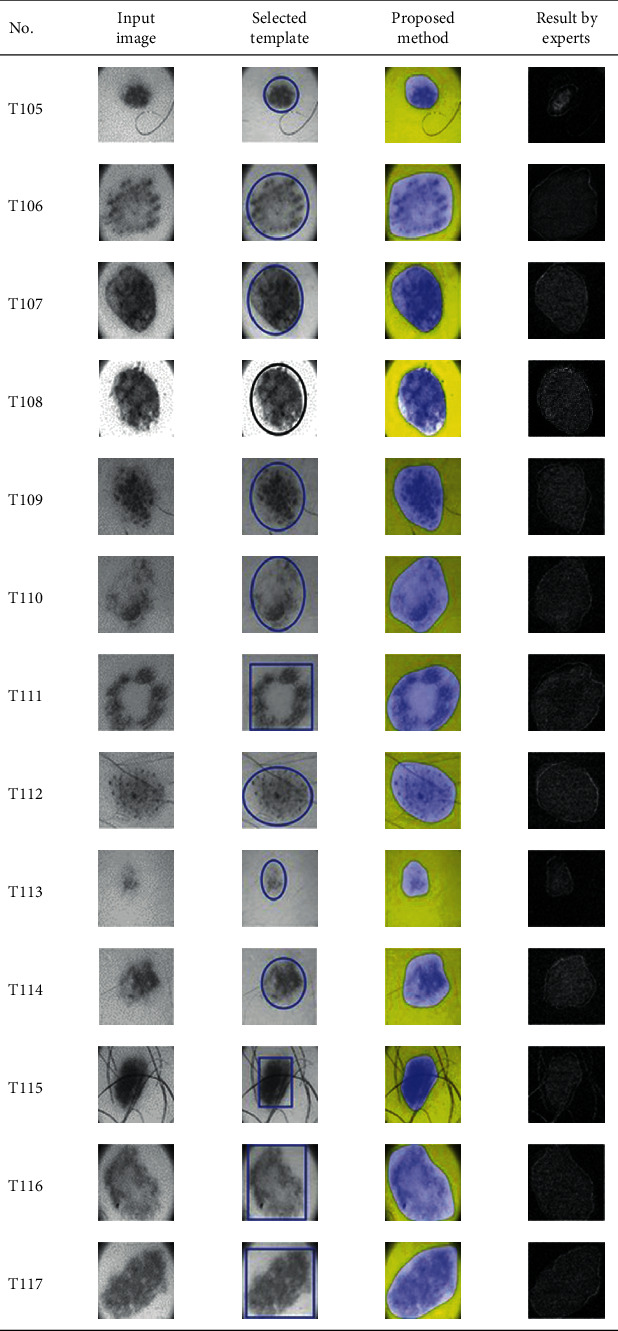
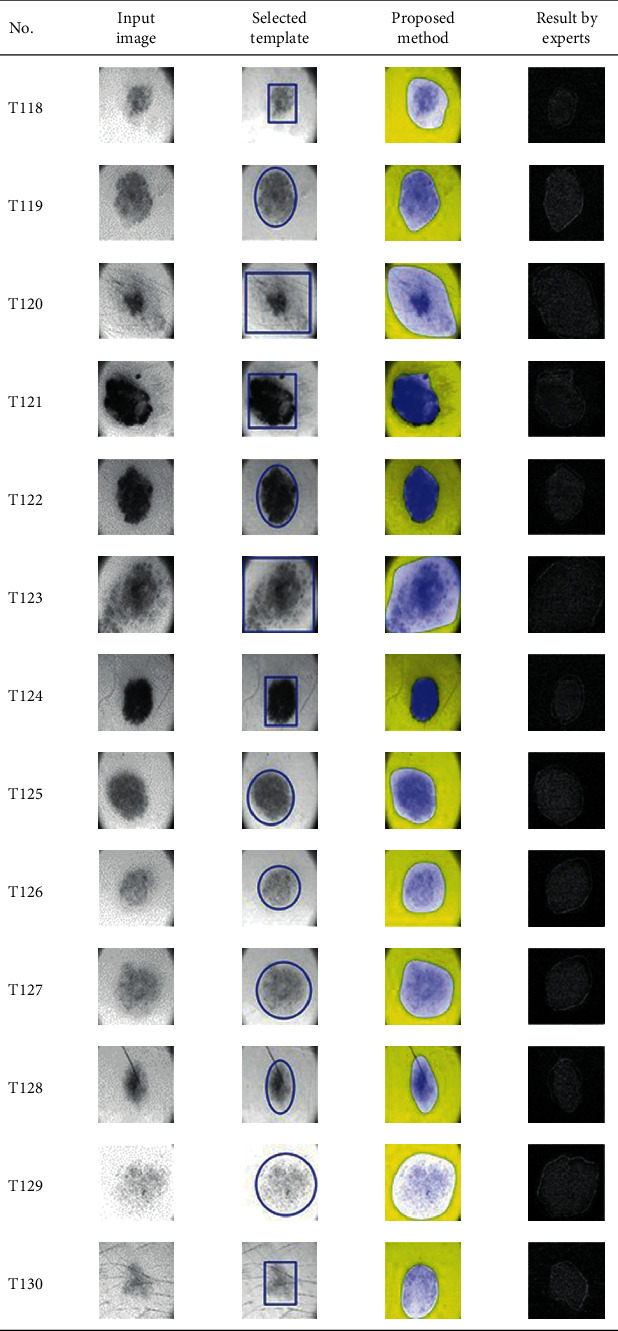
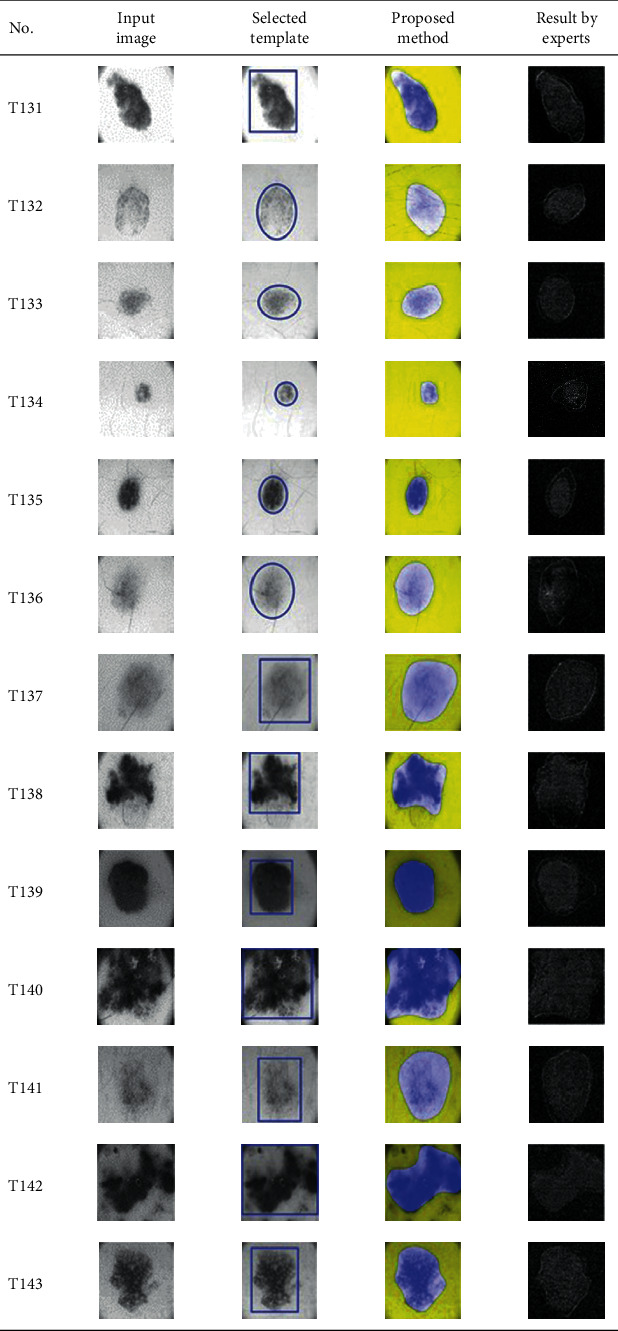
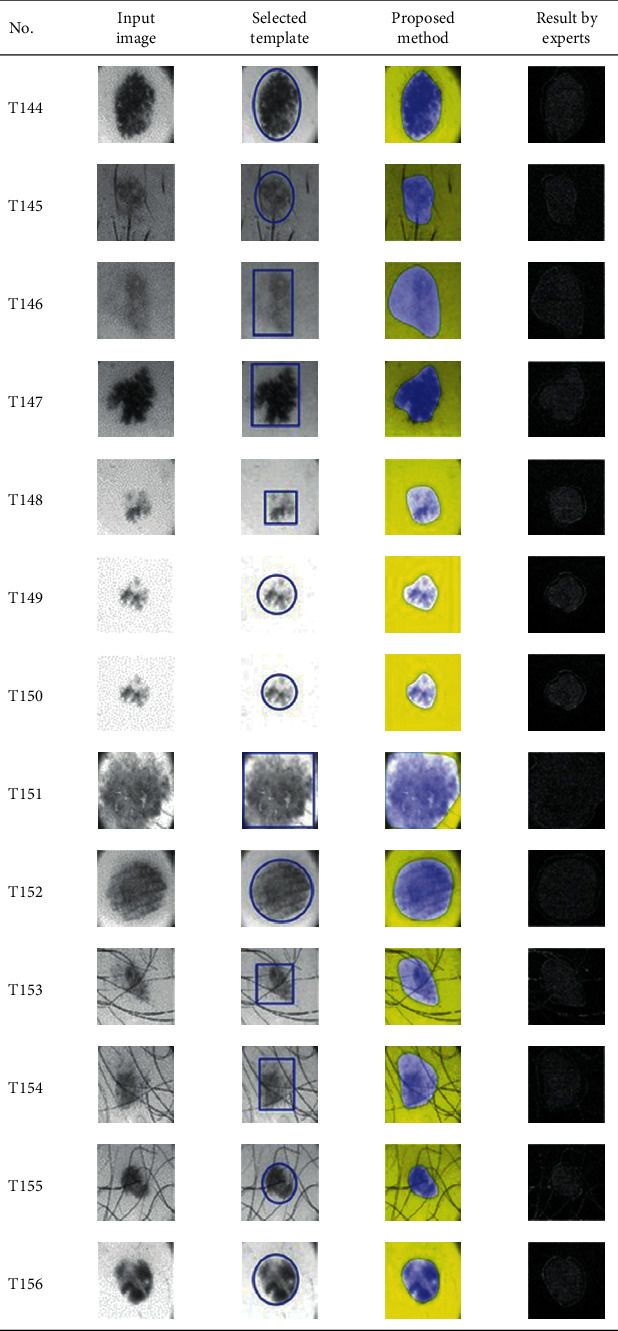
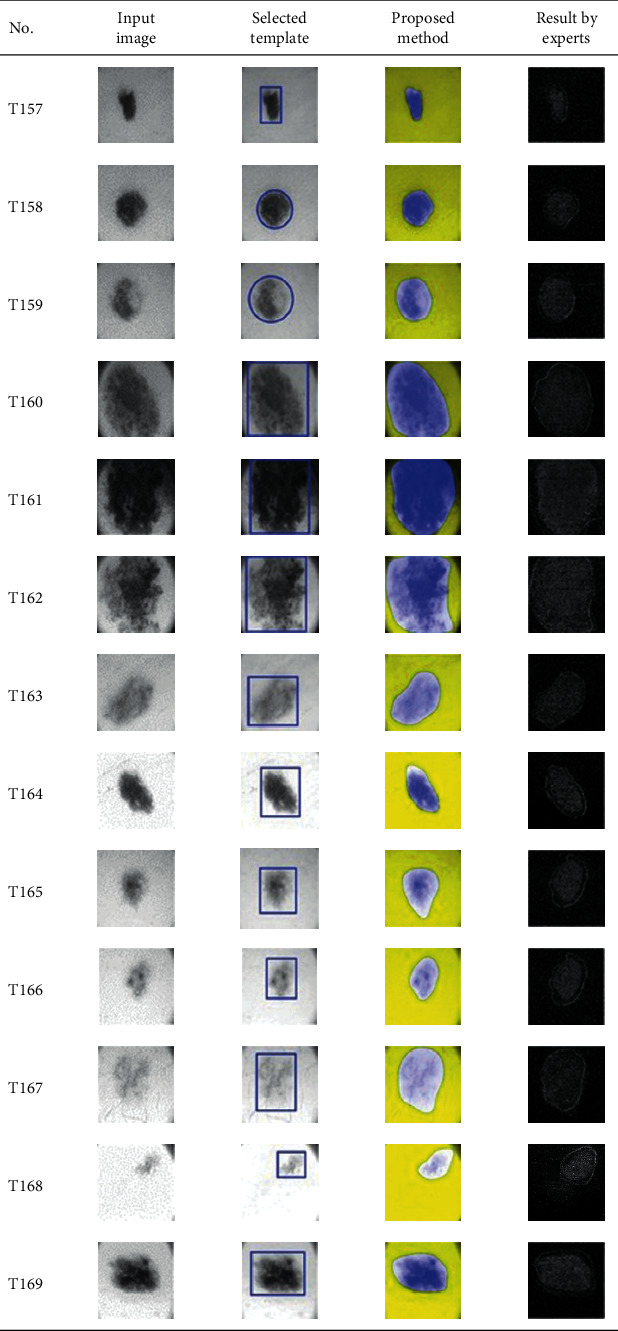
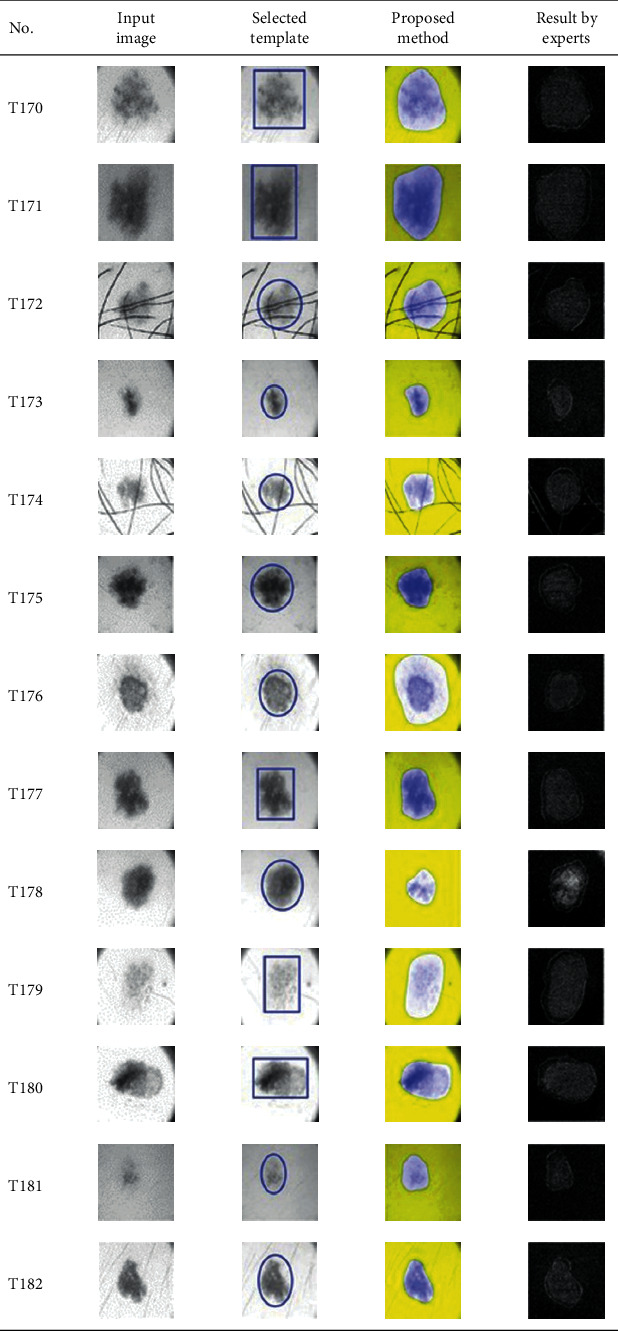
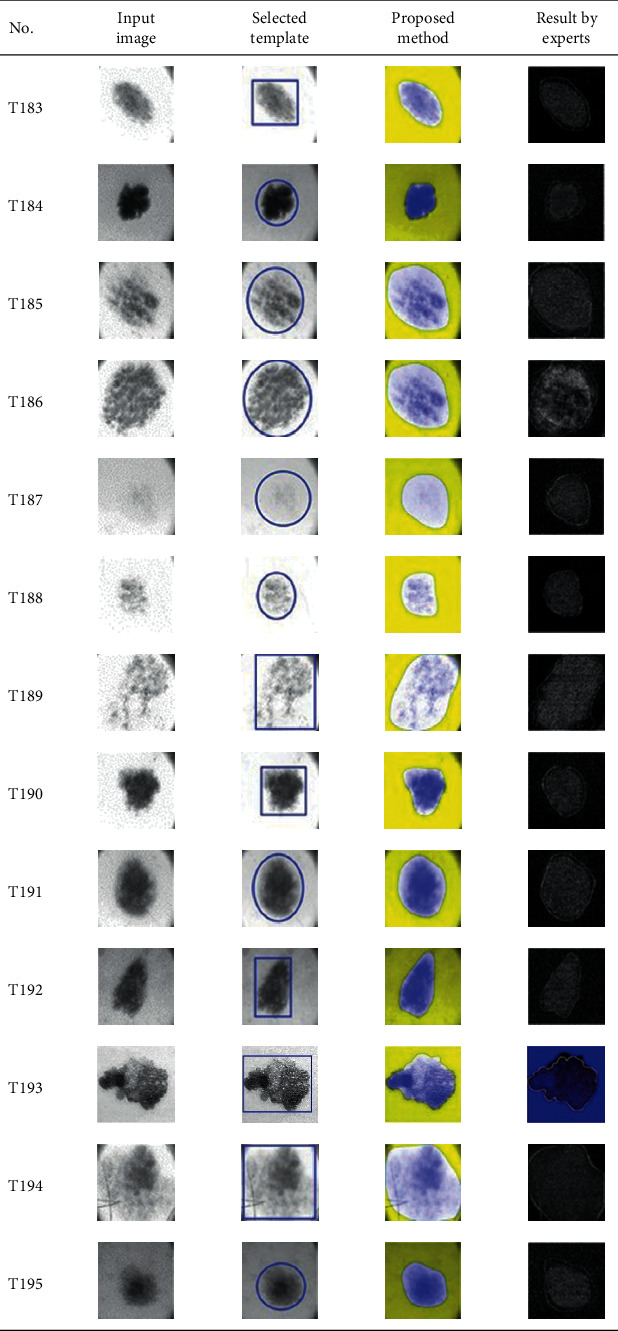
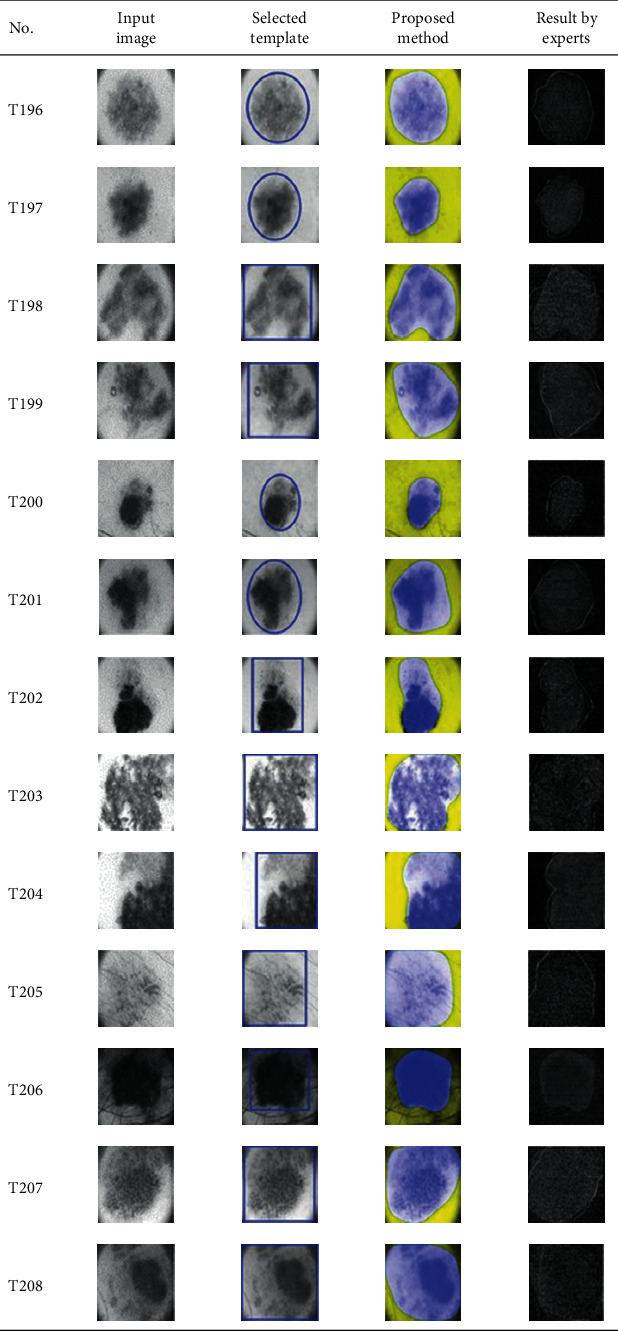
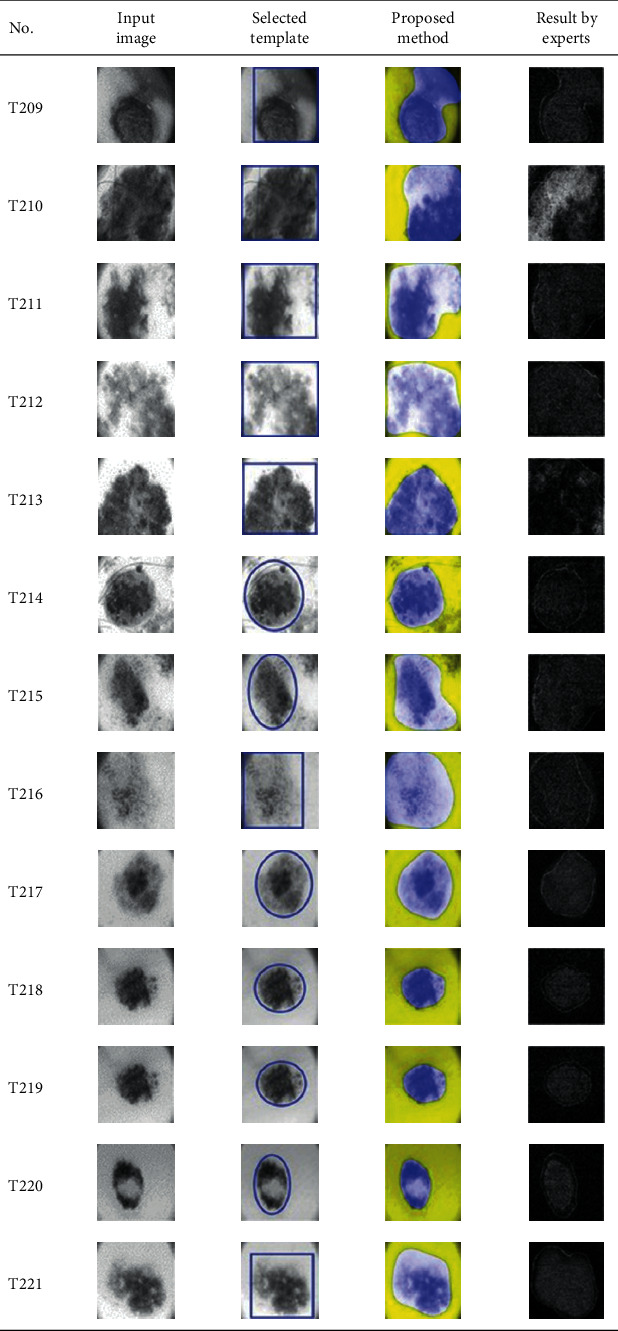
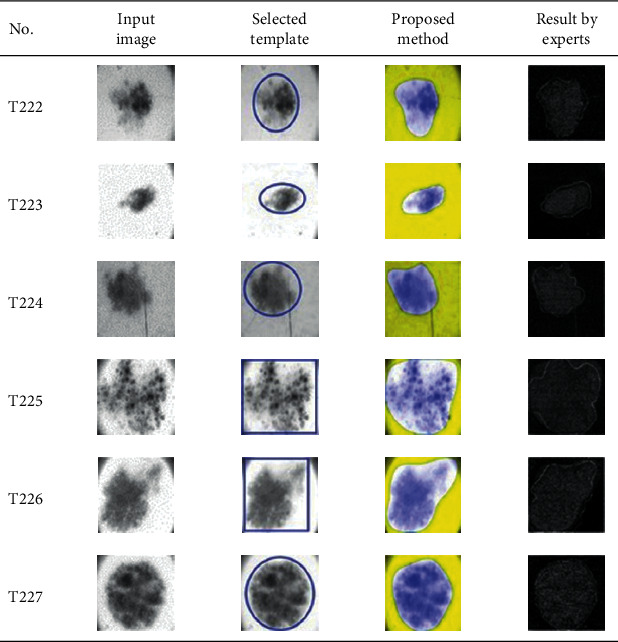

**Table 3:** Specification of experiment set up.

Image	Size	256 × 256 pixels
	Gray level	256
	No. of cancer boundaries per image	1
Program	Basic programing	MATLAB_R2018b
	Segmentation	Snake internal force matrix 2D, snake internal force matrix 3D, snake move iteration 2D, snake move iteration 3D, snake 2D, snake 3D
	Computer	Device name: LAPTOP-ENCF7NAD Processor: Intel(R) Core(TM) i5-1035G1 CPU@1.00 GHz 1.19 GHz Installed RAM: 8.00 GB (7.78 GB usable) Product ID: 00327-35165-87873-AAOEM System type: 64-bit operating system, x64-based processor

**Table 4:** A sample of parameter sets selected by the proposed method.

Parameter	Interaction	nPoints	Sigma1	Sigma2	Sigma3	Wline	Wedge	Wterm	Kappa	Alpha	Beta
*Parameter set for circular template*											
	1000	800	5	5	0	0	10	50	2	3	0.001

*Parameter set for the ellipse template*											
	1000	100	5	5	0	0	10	100	2	2	0.01

*Parameter set for the rectangular template*											
	1000	1000	5	5	0	0	10	150	2	3	0.01

**Table 5:** Comparison between conventional methods and the proposed method.

Model	Average accuracy (%) Region-based measurement			
	JAC	SEN	SPE	ACC
Model ESM [13]	—	85.13	99.83	96.41
Model ESM/AC [14]	—	97.07	99.61	99.20
Proposed method	97.35	97.43	99.87	99.46

## Data Availability

The data used to support the findings of this study are available from the corresponding author upon request.
